# Molecular Subtypes of Extra-pulmonary Neuroendocrine Carcinomas Identified by the Expression of Neuroendocrine Lineage-Specific Transcription Factors

**DOI:** 10.1007/s12022-022-09722-4

**Published:** 2022-05-24

**Authors:** Jasna Metovic, Anna La Salvia, Ida Rapa, Francesca Napoli, Nadia Birocco, Maria Pia Bizzi, Rocio Garcia-Carbonero, Libero Ciuffreda, Giorgio Scagliotti, Mauro Papotti, Marco Volante

**Affiliations:** 1 Department of Oncology, University of Turin; Pathology Unit at Città della Salute e della Scienza Hospital, via Santena 7, Turin, Italy; 2 Division of Medical Oncology, Hospital Universitario, 12 de Octubre, Madrid, Spain; 3 Department of Oncology, University of Turin; Pathology Unit at San Luigi Hospital, Regione Gonzole 10, 10043 Orbassano, Turin, Italy; 4 Medical Oncology Unit, Città Della Salute e Della Scienza Hospital, Turin, Italy; 5 Medical Oncology Unit, San Luigi Hospital, Orbassano, Turin, Italy; 6 Department of Oncology, University of Turin; Medical Oncology Unit at San Luigi Hospital, Orbassano, Turin, Italy

**Keywords:** Extra-pulmonary neuroendocrine carcinoma, Transcriptional profile, Classification, *INSM1*, Prognosis

## Abstract

Extra-pulmonary neuroendocrine carcinomas (EPNEC) represent a group of rare and heterogenous neoplasms with adverse clinical outcome. Their molecular profile is largely unexplored. Our aim was to investigate if the major transcriptional drivers recently described in high-grade pulmonary neuroendocrine carcinomas characterize distinct molecular and clinical subgroups of EPNEC. Gene expression of *ASCL1*, *NEUROD1*, *DLL3*, *NOTCH1*, *INSM1*, *MYCL1*, *POU2F3*, and *YAP1* was investigated in a series of 54 EPNEC (including 10 cases with mixed components analyzed separately) and in a group of 48 pulmonary large cell neuroendocrine carcinomas (P-LCNEC). Unsupervised hierarchical cluster analysis classified the whole series into four major clusters. P-LCNEC were classified into two major clusters, the first *ASCL1/DLL3/INSM1-*high and the second (including four EPNEC) *ASCL1/DLL3*-low but *INSM1*-high. The remaining EPNEC cases were sub-classified into two other clusters. The first showed *INSM1*-high and alternative *ASCL1/DLL3* or *NEUROD1* high expression. The second was characterized mainly by *MYCL1* and *YAP1* overexpression. In the ten cases with mixed histology, *ASCL1*, *DLL3*, *INSM1*, and *NEUROD1* genes were significantly upregulated in the neuroendocrine component. Higher gene-expression levels of *NOTCH1* and *INSM1* were associated with lower pT stage and negative nodal status. Low *INSM1* gene expression was associated with shorter overall survival in the entire case series (*p* = 0.0017) and with a trend towards significance in EPNEC, only (*p* = 0.06). In conclusion, our results show that EPNEC possess distinct neuroendocrine-lineage-specific transcriptional profiles; moreover, low *INSM1* gene expression represents a novel potential unfavorable prognostic marker in high-grade NECs including those in extra-pulmonary location.

## Introduction

In recent years, small cell lung carcinoma (SCLC) has been sub-classified both at the genomic and transcriptional level into molecular subgroups associated with different expression of neuroendocrine markers and to a potentially different pathogenesis. Major transcriptional drivers were initially indicated in *ASCL1* and *NEUROD1* with an apparent mutually exclusive activity in the regulation of neuroendocrine differentiation [1]. More recently, alternative transcriptional patterns have been identified in SCLC, and four major clusters were defined by the preferential expression of *ASCL1*, *NEUROD1*, *POU2F3*, or *YAP1* genes [2].

Data on pulmonary large cell neuroendocrine carcinomas (P-LCNEC) are more limited. They share some genomic alterations with adenocarcinomas and squamous cell carcinomas [3]. However, likewise SCLC, P-LCNEC have been classified into two molecular subgroups, the *ASCL1*/*DLL3-*high and *NOTCH1-*low and the *ASCL1*/*DLL3-*low and *NOTCH1*-high subtypes [4]. The expression of other transcriptional regulators in P-LCNEC was not explored in detail, so far.

Extra-pulmonary neuroendocrine carcinomas (EPNEC) are much rarer than their pulmonary counterpart, being 1/10 of all neuroendocrine carcinomas [5]. Chemotherapy regimens are similar to those adopted in SCLC patients [6], with median overall survival of 14.9 months in recent studies [7]. EPNEC are sub-classified into small cell and large cell types [8]. Large cell type is associated with lower Ki-67 index, reduced sensitivity to first-line platinum/etoposide treatment, and shorter progression-free and overall survivals [9]. The genetics of EPNEC remain poorly understood. Available data are limited by the small number of cases analyzed in each study and the heterogeneity of primary locations. *TP53* followed by *RB1* alterations are the most prevalent drivers in EPNEC irrespective of the primary site. The prevalence of other molecular alterations reflects the similarity with site-specific non-neuroendocrine carcinomas, such as *KRAS*, *BRAF*, and *APC* mutations in gastroenteropancreatic neuroendocrine carcinomas (GEP-NECs) and *TERT* promoter mutations in bladder cancer [8, 10, 11]. The gene expression of neuroendocrine lineage-specific transcriptional markers has never been investigated in EPNEC, so far. Some of the transcriptional regulators mentioned above have been investigated at the protein level (i.e., *ASCL1* and *INSM1* protein—hASH1 and INSM1—products) as immunohistochemical markers of neuroendocrine differentiation [12–15].

Finally, EPNEC may occur as combined carcinomas having a more or less extensive neuroendocrine component admixed with a conventional exocrine component (of the glandular, squamous or urothelial type). Apart from few genomic studies showing almost stable genotypes of the two components [16, 17], the molecular mechanisms acting in the bi-directional clonal evolution of these tumors are largely unexplored. Therefore, it might be supposed that they could be associated with a differential expression of neuroendocrine-specific lineage genes.

Based on the aforementioned data, the aims of the present study were to evaluate the expression of key regulators of neuroendocrine differentiation (*ASCL1*, *NEUROD1*, *DLL3*, *NOTCH1*, *INSM1*, *MYCL1*, *POU2F3*, and *YAP1*) in a series of EPNEC (both pure and mixed) from a variety of sites as well as in P-LCNEC, and to correlate their molecular signatures with pathological and clinical parameters.

## Materials and Methods

### Case Series

Fifty-four cases of EPNEC were retrieved from the pathology files of the San Luigi (Orbassano, Turin, Italy) and at the “Città della Salute e della Scienza” (Turin, Italy) University Hospitals. A series of 48 P-LCNEC was also collected from the same Institutions. All tissue samples were anonymized by a staff member of the Pathology Department not involved in the study. The study was approved by the Institutional Review Board of the hospital (Ethics Committee Approval no. 167/2015-prot.17975, October 21, 2015). Eligibility criteria were as follows: (a) a confirmed histological diagnosis after blind revision by two of us (MV and MP) following the appropriate WHO classifications [18–21] and (b) availability of residual paraffin material for molecular analysis. In cases of unknown primary tumor, the 2019 WHO classification of tumors of the digestive system was adopted. Whenever available, immunohistochemical markers performed at the time of diagnosis were re-evaluated. In cases with incomplete baseline immunohistochemical assessment, epithelial (pan-cytokeratin cocktails) and neuroendocrine markers (chromogranin A, synaptophysin, and/or INSM1 protein) were performed according to standard protocols in use for diagnostics. All enrolled cases had at least two positive neuroendocrine markers, as suggested by the upcoming WHO Classification of Endocrine and neuroendocrine tumors [22]. Moreover, all but three cases (all three in the EPNEC group, showing lack of positivity in internal control cells) had Ki-67 data available, either from revision of archival slides or after new staining procedures. All pathological and clinical information available were also collected. All P-LCNEC were surgically resected specimens. By contrast, 21 EPNEC cases were large biopsies or metastasectomies; therefore, pT and pN stages were missing. Survival data were available for 39 EPNEC and 44 P-LCNEC.

### RNA Extraction From Formalin-fixed Paraffin-embedded Tissues and Gene Expression Analyses

Ten-micrometer-thick sections were cut in RNase-free conditions from paraffin-embedded tissues following microdissection using a scalpel at a magnification of 100 × from hematoxylin–eosin stained slides. In 10 cases with mixed neuroendocrine and non-neuroendocrine components, the two populations were separately dissected and analyzed. Total RNA isolation was performed by commercially available RNA extraction kits designed for paraffin material according to the manufacturer’s instructions (miRNeasy FFPE kit; Qiagen, Hilden, Germany).

RT reactions were performed using 10 ng total RNA in a volume of 15 µl at the following conditions: 16 °C for 30 min, 42 °C for 30 min, 85 °C for 5 min, and 4 °C for 5 min. Expression levels of all genes studied and internal reference were examined using a fluorescence-based real-time detection method (ABI PRISM 7900 Sequence Detection System—Taqman; Applied Biosystems, Foster City, CA). The following TaqMan gene expression assays (Applied Biosystems) were used according to the manufacturer’s instructions: *ASCL1* (HS00269932_m1), *DLL3* (HS01085096_m1), *INSM1* (Hs00357871_s1), *MYCL1* (Hs00420495_m1), *NEUROD1* (HS01922995_s1), *NOTCH1* (Hs01062014_m1), *POU2F3* (Hs00205009_m1), and *YAP1* (Hs00902712_g1). The *ACTB* (Hs01060665_g1) assay served as references for gene analyses.

Each measurement was performed in duplicate. The ΔΔCt values were calculated subtracting ΔCt values of sample and ΔCt value of Stratagene (a pool of RNA derived from normal different tissues; Stratagene, CA) and converted to ratio by the following formula: 2^−ΔΔCt^.

### Statistical Analyses

Rows and columns were clustered using the hierarchal clustering tool in Morpheus (https://software.broadinstitute.org/morpheus/documentation.htm) using the one minus Pearson correlation matrix and the average linkage method. The log2 fold change values were *z*-score adjusted before clustering. Correlation among gene expression was assessed by means of Spearman’s correlation test. To obtain a graphic representation of the interactions among the molecules investigated, the STRING database was used (https://string-db.org/). Mann–Whitney test was used to test the association between gene expression and clinical pathological variables, as appropriate. Overall survival endpoint was defined as the time between diagnosis and patients’ death. Univariate analysis was performed with Kaplan–Meier curve estimation and the significance was verified by the log-rank test. Median values were used as cut offs for low and high gene expression. Multivariate analysis was performed using a Cox proportional hazard model. All analyses were performed using GraphPad software (Graphpad Software Inc., La Jolla, CA) and SPSS software (IBM corporation, Armonk, USA). A *p* value lower than 0.05 was considered statistically significant in all analyses.

## Results

### EPNEC Locations

**Fig. 1:**
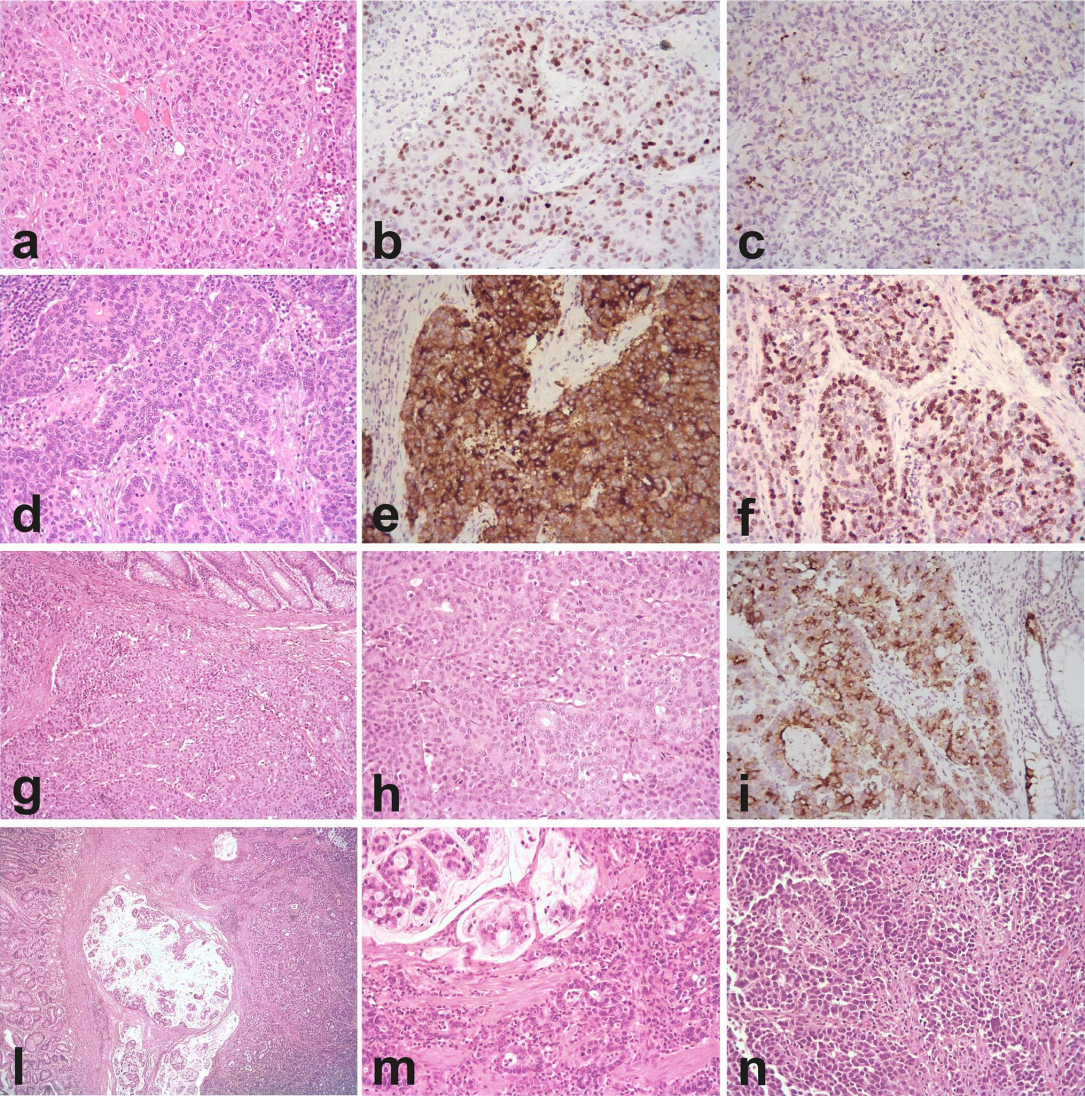
Representative EPNEC cases from the series investigated. **a**–**c** EPNEC of large cell type, unknown primary, brain metastasis, positive for INSM1 protein (**b**) and chromogranin A (**c**), with focal dot-like pattern. **d**, **e** EPNEC of large cell type, unknown primary, lymph node metastasis, positive for synaptophysin (**e**) and with high Ki-67 index (**f**). **g**–**i** EPNEC, large cell type primary of the colon, positive for chromogranin A (**i**). **l**, **m** Mixed neuroendocrine/non-neuroendocrine neoplasm of the duodenum with adenocarcinoma component with mucin production (**l** and **m**) and small cell carcinoma component (**n**) (all original magnifications 200 × , except g 100 × and l 40 ×)

The major clinical and pathological features of the cases analyzed are summarized in Table 1. In the gastroenteropancreatic group, 11 cases were from the colon, four cases, each, from stomach and esophagus, three cases from the duodenum, two cases from the anal canal, and one case, each, from the pancreas and ileum. In the genitourinary group, all cases were from the bladder, except for two cases from the renal pelvis and one case, each, from the cervix and ovary. The primary origin of the remaining 6 cases was unknown. Of the ten cases with mixed neuroendocrine and non-neuroendocrine histology, 6 were from the bladder (with an urothelial carcinoma component) and four were from the gastrointestinal tract (two from the duodenum, one from the left colon, and one from the stomach, all with an adenocarcinoma component). Representative cases from our series are illustrated in Fig. 1.

**Table 1:** Major clinical and pathological features of the cases investigated

Parameter	EPNEC [#54]	P-LCNEC [#48]
Sex (M/F)	36/18	40/8
Age, median (range)	73 (37–88)	67 (48–82)
*Location*		
GEP system	26	/
GU tract	22	/
unknown	6	/
*NEC Histology*		
Small cell type (%)	26 (48%)	/
Large cell type (%)	28 (52%)	48
Mixed non-NE component (%)	10 (18%)	/
Stage T3–4 (%)	25 (75%)*	15 (31%)
Positive nodal status (%)	16 (57%)**	13 (27%)
Ki-67, mean %	70	57
Ki-67 > 55% (%)	40 (78%)***	30 (62%)
Died of disease (%)	27 (69%)****	20 (45%)^*****^
Mean overall survival, months	21	40

*GEP*, gastro-entero-pancreatic; *GU*, genito-urinary; *EPNEC*, extra-pulmonary neuroendocrine carcinoma; *P-LCNEC*, pulmonary large cell neuroendocrine carcinoma; *NE*, neuroendocrine; *DOD*, died of disease

*21 missing; **26 missing; ***3 missing; ****15 missing; *****4 missing

### Gene Expression Patterns

A strong reciprocal positive correlation was observed in the whole series between *INSM1*, *ASCL1*, and *DLL3* gene expression. *INSM1* gene expression was also positively associated with *NOTCH1*. *MYCL1* was strongly negatively correlated with *NOTCH1* and *INSM1*. *POU2F3* was inversely correlated with *ASCL1* and *DLL3*, whereas *YAP1* was inversely correlated with *ASCL1* (Table 2).

**Table 2:** Reciprocal correlation among the genes tested

	***ASCL1***	***DLL3***	***NEUROD1***	***INSM1***	***POU2F3***	***MYCL1***	***YAP1***
*NOTCH1*	*R*: −0.17 *p*: 0.07	*R*: 0.25 *p*: 0.006	*R*: −0.23 *p*: 0.01	*R*: 0.45 *p*: < 0.0001	*R*: 0.06 *p*: 0.49	*R*: −0.48 *p*: < 0.0001	*R*: 0.27 *p*: 0.003
*ASCL1*	-	*R*: 0.67 *p*: < 0.0001	*R*: 0.15 *p*: 0.11	*R*: 0.40 *p*: < 0.0001	*R*: −0.25 *p*: 0.007	*R*: 0.18 *p*: 0.06	*R*: −0.49 *p*: < 0.0001
*DLL3*	-	-	*R*: 0.05 *p*: 0.59	*R*: 0.60 *p*: < 0.0001	*R*: −0.30 *p*: 0.0014	*R*: −0.11 *p*: 0.24	*R*: −0.21 *p*: 0.02
*NEUROD1*	-	-	-	*R*: 0.16 *p*: 0.10	*R*: −0.12 *p*: 0.19	*R*: 0.09 *p*: 0.35	*R*: −0.18 *p*: 0.06
*INSM1*	-	-	-	-	*R*: −0.17 *p*: 0.06	*R*: −0.32 *p*: 0.0006	*R*: −0.22 *p*: 0.02
*POU2F3*	-	-	-	-	-	*R*: 0.22 *p*: 0.02	*R*: 0.17 *p*: 0.06
*MYCL1*	-	-	-	-	-	-	*R*: −0.08 *p*: 0.40

*R*, Spearman’s correlation value

**Fig. 2:**
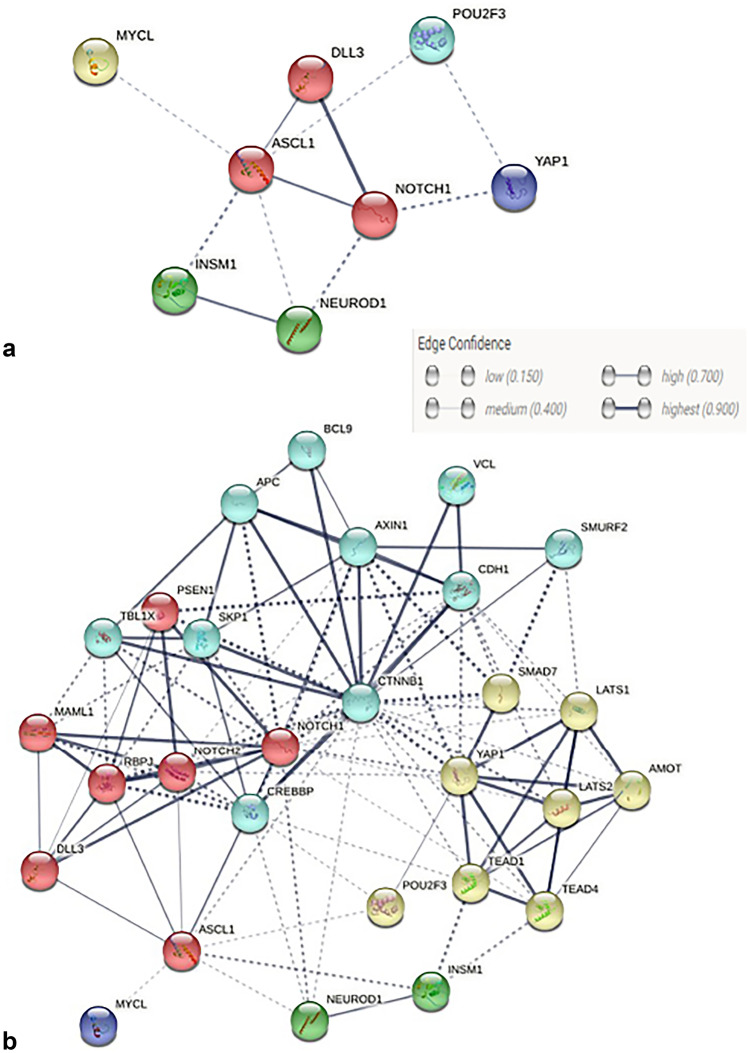
Representation of the interaction between investigated molecules (**a**) and their most significant partners (**b**) visualized by STRING software. Black line type and saturation of the edges represent the confidence score of the functional association

The definition of functional association networks among the molecules investigated and their most relevant partners showed that *ASCL1*, *DLL3*, and *NOTCH1* are functionally associated each other and significantly interact with the *CTNBB1* pathway (Fig. 2). By contrast, *INSM1/NEUROD1*, *YAP1*, *POU2F3*, and *MYCL1* belong to independent pathways, not reciprocally associated.

**Fig. 3:**
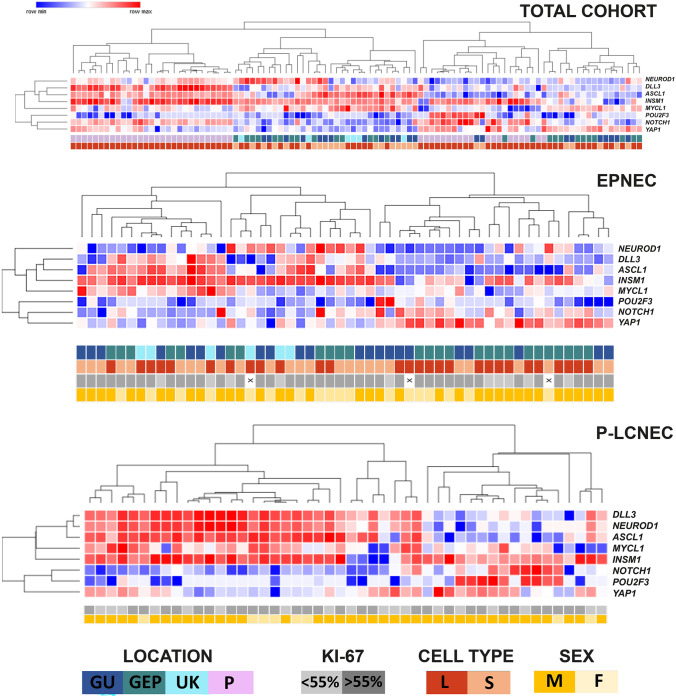
Unsupervised cluster analysis of the entire cohort and in P-LCNEC and EPNEC subgroups based on gene expression patterns. GEP, gastro-entero-pancreatic system; GU, genito-urinary tract; UK, unknown; P, pulmonary, L, large; S, small; M, male; F, female

By means of unsupervised cluster analysis, patterns of gene expression were able to stratify the cases into four major subgroups (Fig. 3). The first cluster was composed exclusively of P-LCNEC and was characterized by gene overexpression of *ASCL1*, *DLL3*, and *INSM1*. The second cluster was composed exclusively of EPNECs and mirrored in part the first cluster. It was characterized by *INSM1* gene overexpression and alternative overexpression of *ASCL1* and *DLL3* or *NEUROD1*. The third cluster was more heterogeneous and included 18 cases of P-LCNEC and 4 cases of EPNEC. It was characterized by low gene expression of *ASCL1* and *DLL3* and overexpression of *INSM1*, with variable expression of *POU2F3*, *NOTCH1*, and *YAP1*. The fourth cluster was composed of EPNEC cases, except for one case of P-LCNEC, and was characterized mainly by *MYCL1* and *YAP1* overexpression and low expression of all other genes.

P-LCNEC and EPNEC were also analyzed separately. In P-LCNEC, high *INSM1* gene expression characterized the two main clusters. In addition, the first cluster showed overexpression of *ASCL1*, *DLL3*, and *NEUROD1*, whereas the second cluster showed overexpression of *YAP1*, *POU2F3*, and *NOTCH1*. In EPNEC, one main cluster was dominated by *INSM1* gene overexpression and by the alternative expression of *ASCL1-DLL3* or *NEUROD1*. These latter markers were downregulated in the second main cluster, which was dominated by *YAP1* and—to a lower extent—*NOTCH1* overexpression. In all cluster analyses, molecular subgroups were not associated with the clinical or pathological parameters reported in Fig. 3, except for unknown primary site in EPNEC that was exclusively represented in the *INSM1*-high/*ASCL1-DLL3* or *NEUROD1*-high cluster (Fisher test, *p* = 0.028).

### Clinical and Pathological Correlations

The eight genes analyzed separately were variably distributed in the different locations (Table 3). Significantly different site-specific expression levels were observed for all genes, except for *NEUROD1* and *POU2F3*. In particular, P-LCNEC displayed higher gene expression of *NOTCH1*, *DLL3*, *YAP1*, and *INSM1* and lower gene expression of *MYCL1*, as compared to the other locations. Neuroendocrine carcinomas from the genitourinary tract had the highest levels of *ASCL1* and *MYCL1*. In EPNEC, the cell type was not significantly associated with gene expression levels, except for a higher gene expression of *ASCL1* and a lower gene expression of *YAP1* in small cell type. Higher gene expression levels of both *NOTCH1* and *INSM1* were strongly associated with lower pT stage and negative nodal status. High pT stage was associated with high gene expression of *ASCL1* and low gene expression of *DLL3*. Low expression of *YAP1* was associated with positive nodal status.

**Table 3:** Correlation of gene expression with major clinical and pathological variables

		***NOTCH1***	***ASCL1***	***DLL3***	***NEUROD1***	***INSM1***	***POU2F3***	***MYCL1***	***YAP1***
**Location**	Lung	6.744	31.28	118.9	10.99	128,721	10.66	33.09	0.4887
	GEP	0.4079	23.30	3.00	144.2	6618	0.3908	204.5	0.2871
	GU	0.3963	144.5	5.742	57.03	6281	12.54	554.1	0.2368
	Unknown	0.1500	121.3	10.35	38.78	13,629	0.3375	151.8	0.1020
	*P value*	< *0.0001*	*0.02*	< *0.0001*	*0.11*	< *0.0001*	*0.697*	< *0.0001*	*0.006*
Cell type*	Small	0.3286	128.4	7.082	116.0	7464	10.84	654.9	0.1818
	Large	0.3941	45.33	3.696	88.68	7335	0.3356	153.4	0.2950
	*P value*	*0.11*	*0.016*	*0.14*	*0.57*	*0.67*	*0.76*	*0.35*	*0.007*
**pT stage**	pT1-2	5.014	40.84	103.6	59.68	82,231	8.828	384.1	0.3323
	pT3-4	2.221	51.50	26.62	43.68	63,909	4.116	248.7	0.4005
	*P value*	*0.002*	*0.03*	*0.017*	*0.96*	*0.007*	*0.50*	*0.17*	*0.76*
**pN stage**	pN0	6.123	33.49	99.35	12.51	101,191	12.87	84.15	0.4328
	pN +	1.950	43.75	32.19	110.1	50,266	18.32	78.38	0.2890
	*P value*	*0.0012*	*0.45*	*0.2*	*0.14*	*0.013*	*0.986*	*0.071*	*0.041*

*GEP*, gastro-entero-pancreatic; *GU*, genito-urinary

*EPNEC cases, only

### Gene Expression Levels in Neuroendocrine Vs Non-neuroendocrine Components of Cases With Mixed Histology

**Fig. 4:**
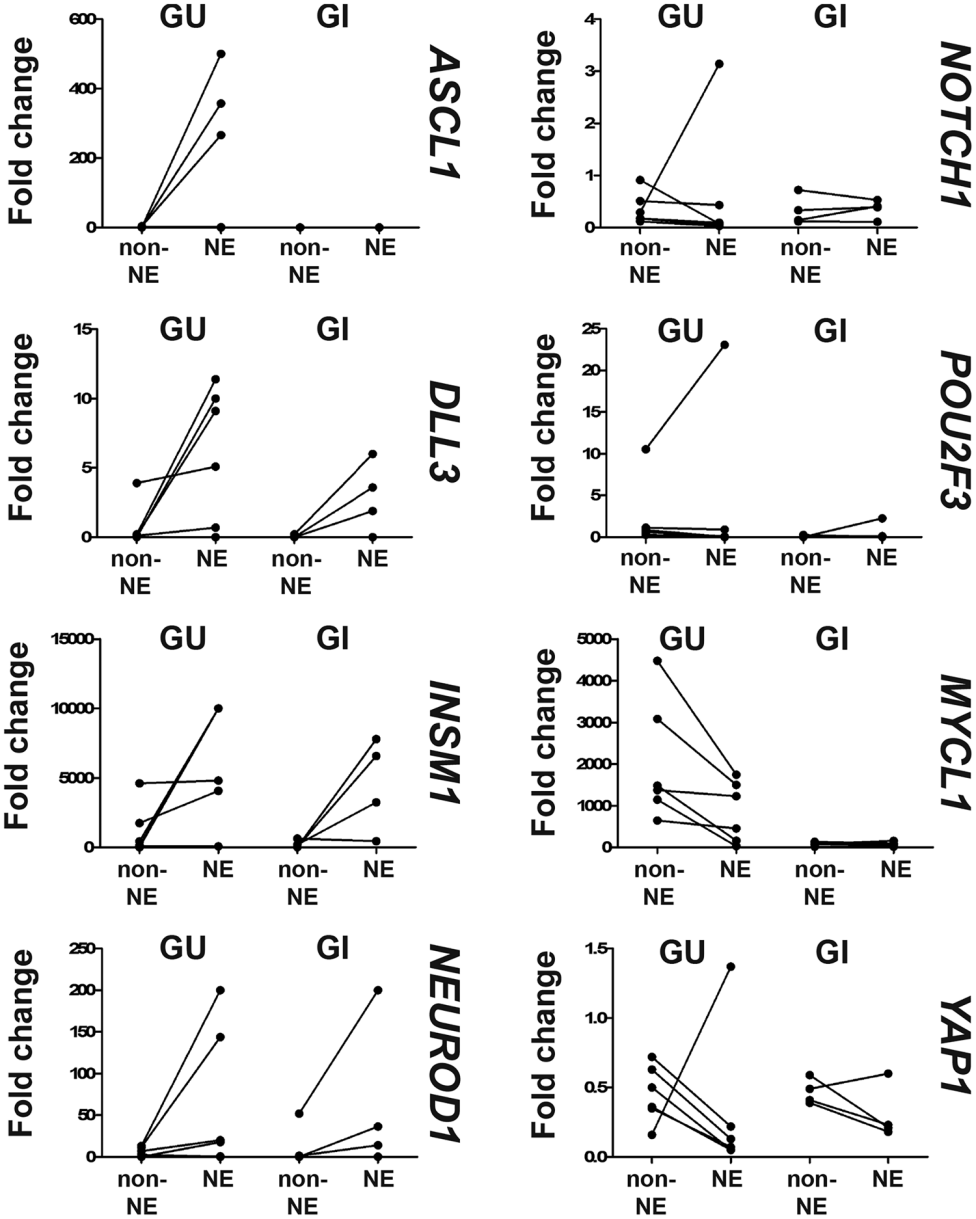
Gene expression patterns in neuroendocrine and non-neuroendocrine microdissected tumor cell populations of ten cases. NE, neuroendocrine; GU, genito-urinary tract; GI, gastrointestinal tract

In ten cases with mixed histology, the expression of target genes was analyzed independently in neuroendocrine and non-neuroendocrine components (Fig. 4). In most cases, the neuroendocrine component displayed upregulation of *ASCL1*, *DLL3*, *INSM1*, and *NEUROD1* genes as compared to the non-neuroendocrine population. This result was independent from the tumor location for all genes, except for *ASCL1* that displayed a differential profile of expression only in cases of the genitourinary tract. By contrast, *NOTCH1*, *POU2F3*, *MYCL1*, and *YAP1* did not show significant modifications (except for single cases) of their gene expression in the neuroendocrine vs non-neuroendocrine components. Indeed, the two latter genes showed even a trend to down regulation in the neuroendocrine as compared to the non-neuroendocrine populations.

### Survival Analyses

**Fig. 5:**
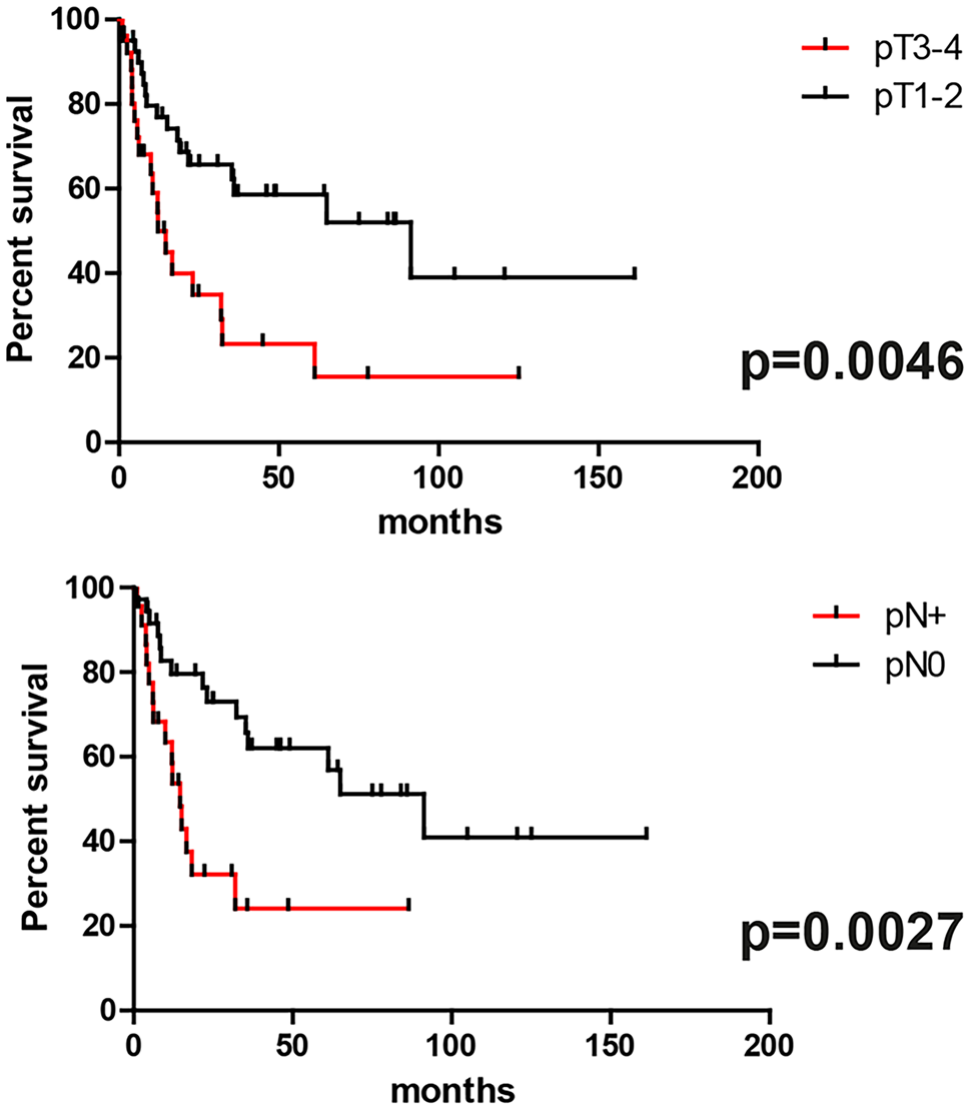
Overall survival curves in the entire cohort according to T stage and nodal status

**Fig. 6:**
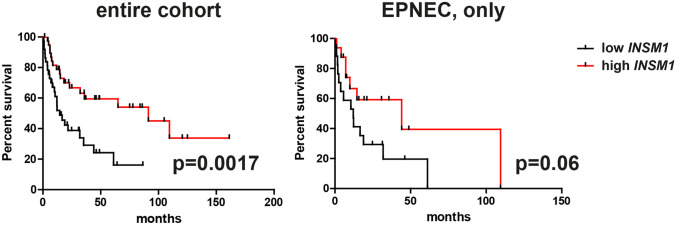
Overall survival curves in the entire cohort and in EPNEC, only, according to *INSM1* expression (low and high as determined by median gene expression values, see text)

By means of univariate overall survival analyses in the whole population, pT3-4 stage and positive nodal status were associated with significantly shorter survivals (Table 4) (Fig. 5). *INSM1* was the single gene significantly associated with survival, with low gene expression levels associated with shorter overall survival in the whole population and with a trend towards significance in the EPNEC group (not shown in Table 4; median survival: 12.10 vs 44.10 months, hazard ratio 2.22, confidence intervals 0.94–5.25, *p* = 0.06) (Fig. 6). At multivariable analysis in the whole population, pN stage, only, retained statistical significance (coefficient 0.77, *p* = 0.0169).

**Table 4:** Univariate survival analysis

Parameter	Median survival (months)	HR [CI]	*p*
Cell type (small vs large)**	14.5–16.6	1.233 [0.51–2.99]	0.6441
pT stage (pT3-4 vs pT1-2)	12.2 vs 91.3	2.88 [1.39–6.0]	0.0046
pN stage (pN + vs pN0)	14.6 vs 91.3	3.58 [1.55–8.26]	0.0027**
*NOTCH1* low vs high*	44.1 vs 75.0	1.084 [0.53–2.20]	0.82
*ASCL1* low vs high*	31.9 vs 44.1	1.4 [0.76–2.58]	0.28
*DLL3* low vs high*	35.3 vs 32.3	1.05 [0.38–1.29]	0.87
*NEUROD1* low vs high*	31.9 vs 44.1	0.95 [0.52–1.75]	0.88
*INSM1* low vs high*	14.5 vs 91.3	2.808 [1.47–5.35]	0.0017
*YAP1* low vs high*	18.3 vs 61.2	1.25 [0.67–2.32]	0.48
*POU2F3* low vs high*	31.9 vs 44.1	1.0 [0.55–1.84]	0.99
*MYCL1* low vs high*	61.2 vs 31.9	0.69 [0.38–1.29]	0.25

*HR*, hazard ration; *CI*, confidence Intervals

*according to the median level; **extra-pulmonary neuroendocrine carcinomas, only

## Discussion

In the present study, we analyzed the expression of a panel of transcriptional regulators of neuroendocrine differentiation in EPNEC as compared to a series of P-LCNEC.

The project stemmed from the growing evidence of the impact of lineage-specific transcription factors (including *INSM1*, *YAP1*, *POU2F3*, *MYCL*, and *NEUROD1*) in stratifying SCLC into different molecular subgroups. Our data clearly demonstrated that EPNEC can be classified into molecular transcriptional subclasses partially overlapping those described in SCLC. In parallel, we also tested the gene expression of these transcription factors in P-LCNEC that have incompletely investigated in this respect, so far.

Irrespective of the classification system specific for each location, EPNEC are diagnosed by the presence of an appropriate morphology and the expression of at least one—or better two—neuroendocrine marker, from a panel that includes chromogranin A, synaptophysin, and INSM1 protein [22]. From a pure morphological standpoint, EPNEC of the large or small cell type resemble their pulmonary counterpart, and in the clinical practice they are managed and treated as such. However, some recent genomic data claimed that in the gastroenteropancreatic system they are molecularly closer to the respective adenocarcinoma counterpart than to well-differentiated neuroendocrine tumors [16]. In contrast, the lung genomic [23] and transcriptional [24] data suggest a closer link between high grade neuroendocrine carcinomas (of the small and large cell type) and neuroendocrine tumors, for some authors even within a hypothetical evolutionary context [25].

In this scenario, we might have expected different gene expression patterns of transcriptional regulators in P-LCNEC and EPNEC. Indeed, cluster analysis strongly segregated P-LCNEC from EPNEC, but at the same time these latter were further divided into two clusters that partially mirrored the lung cases. Subgroup analysis of EPNEC cases identified a first cluster characterized by a high *INSM1* expression and by the alternative expression of *ASCL1*/*DLL3* or *NEUROD1*, and a second cluster dominated by *YAP1* and *NOTCH1* overexpression. This global profile is pretty similar to what previously described in SCLC [1, 2]. A similar profile was obtained by subgroup cluster analysis of P-LCNEC. In fact, a first cluster showed an *ASCL1*/*DLL3*/*NEUROD1*-high signature and a second cluster was characterized by preferential expression of *POU2F3*, *NOTCH1*, and/or *YAP1. INSM1* gene over-expression was generally over-represented in P-LCNEC. Correlation analyses of individual genes with location sharply took pulmonary apart from extra-pulmonary cases. In EPNEC, site of origin (except for unknown primary site) and cell type (small vs large) were not influencing their sub-classification. Of note, the distribution of the different extra-pulmonary sites of origin in our series was biased by selection criteria, both related to technical reasons (availability of leftover tumor tissue material and adequacy of RNA extracts for molecular analysis) and to a preeminent load of surgical cases for urological malignancies as compared to gastrointestinal or pancreatic ones.

Cases with mixed neuroendocrine and non-neuroendocrine histology were randomly distributed in the three clusters containing EPNEC cases. Moreover, the modulation of the different genes in neuroendocrine carcinoma components was heterogeneous. In fact, upregulation was evident in neuroendocrine cell populations for *ASCL1*, *DLL3*, *NEUROD1*, and *INSM1* in most cases. All genes were up-modulated in mixed cases irrespective of the tumor location, except for *ASCL1* that was not modulated in mixed cases of the gastrointestinal tract. This latter finding is in agreement with *ASCL1* lower expression in cases from the gastroenteropancreatic system as compared to other locations, as demonstrated in our series and in the literature [12]. In contrast, transcriptional regulators associated to a lower expression of neuroendocrine markers in the SCLC model (in particular *YAP1*, *NOTCH1*, and *POU2F3*) were not generally modulated in neuroendocrine components of mixed cases in our series.

In terms of clinical and pathological correlates, none of the genes investigated has been reported to clearly impact on clinical aggressiveness of SCLC and tested P-LCNEC. *ASCL1* has been found to be associated with lack of EGFR mutations, PD-L1 negative expression, and a poor immune cell infiltration in adenocarcinomas with neuroendocrine differentiation [26], but not to characterize subsets of SCLC with a distinctive clinical outcome.

In our series, *DLL3* was expressed to a higher extent in cases with lower tumor stage. The expression of *DLL3* in EPNEC paves the way to the potential use in these tumors of specific therapies targeting this molecule [27], as also recently suggested for P-LCNEC [28]. Moreover, *DLL3* was identified in a subset of small cell carcinomas of the bladder as a negative prognostic biomarker, and the in vivo efficacy of a *DLL3*-targeting conjugated antibody was demonstrated in a PDX model [29].

Decreased expression of *NOTCH1* was also characteristic in our series of cases with higher pT stage and positive nodal status. This result is possibly explained by the inhibitory effect of the NOTCH pathway on cell growth reported in SCLC cells [30].

INSM1 protein expression has been mostly investigated as a diagnostic immunohistochemical marker for neuroendocrine neoplasms in different organs and settings. However, its comparative expression with other transcriptional regulators of the neuroendocrine phenotype has not been assessed, except for SCLC. Our data confirm that *INSM1* gene is highly expressed in neuroendocrine carcinomas of different sites. With all the possible limitations due to the relatively small sample size and heterogeneity of the case series, *INSM1* low gene expression characterized a subset of cases with more aggressive clinical features and worse outcome. Data on the prognostic role or association with clinical features of *INSM1* gene expression in EPNEC are missing. The adverse prognostic effect of low *INSM1* gene expression described in the present study is partly in contrast with some data available in SCLC of a negative prognostic role of INSM1 protein overexpression [31] and by *INSM1* capability to promote cell growth in vitro [32]. By contrast, a negative prognostic impact of low INSM1 protein expression in SCLC in terms of overall survival and lower rates of response to chemotherapy was reported by McColl and coworkers [33]. Whether *INSM1* gene expression reflects specific genomic profiles or a less differentiated phenotype associated with worse outcome has to be elucidated in future studies. Moreover, due to the lack of robust information, we could not speculate if the prognostic impact of *INSM1* gene expression was attributable to an influence in chemotherapy response.

Undoubtedly, a limitation of our study is the lack of validation of gene expression data through the analysis of protein expression levels using immunohistochemistry. INSM1 protein expression was assessed as a marker of neuroendocrine phenotype in 8 cases, only, thus preventing any possible correlation. Therefore, in a clinical perspective of biomarker testing, our findings should be validated in larger cohorts of samples analyzing protein expression profiles of INSM1 and the other molecules in correlation with clinical and pathological characteristics. Such an approach has been recently proposed in the SCLC model [34].

## Conclusions

In summary, our results show that EPNECs possess distinct neuroendocrine-lineage-specific transcriptional profiles that in part mirror those described for pulmonary small and large cell carcinomas and are independent from the site of origin of the tumor. Decreased gene expression of *INSM1* was associated with characteristics of aggressive disease and shorter overall survival, and *INSM1* potential prognostic role in EPNEC merits validation in larger and independent series.
